# The American Text Book of Operative Dentistry

**Published:** 1898-01

**Authors:** 


### Bibliographical.

THE AMERICAN TEXT BOOK OF OPERATIVE DENTISTRY. In Contributions by Eminent Authorities. Editor: Edward C. Kirk, D. D. S., Professor of Clinical Dentistry in the University of Pennsylvania. Publishers: Lea Brothers & Co., Philadelphia and New York. 1897.

The appearance of a valuable addition to dental literature is always received with pleasure and satisfaction by those who are sincerely interested in the progress of dental science. The American Text Book of Operative Dentistry is such a work, and also one that must add to the reputation of the editor, as well as of those whose labors have assisted him in the preparation of this useful and well written treatise. Consisting of 702 pages of text and 751 fine illustrations, it cannot fail to be a useful study for both student and practitioner.

The contents consist of the following contributions which are well written, and their arrangement worthy of high commendation:

Microscopic Anatomy of the Human Mouth, by Alton Howard Thompson; The Embryology and Histology of the Dental Tissues, by R. R. Andrews; The Examination of Teeth Preliminary to Operation—Methods, Instruments, Appliances—Recording Results, etc., by Louis Jack; Preliminary Preparation of the Teeth—Removal of Deposits and Cleaning of the Teeth—Wedging—Other Methods of Securing Separations—Exposure of Cervical Margins by Slow Pressure, etc., by Louis Jack; Preliminary Preparation of Cavities—Treatment of Hypersensitive Dentine by Sedatives, Obtundents, Local and General Anesthetics—Sterilization, with a brief Consideration of the Physiological and Therapeutic Action of the Medicaments used, by Louis Jack; Preparation of Cavities—Opening the Cavity—Removing the Decay—Shaping the Cavity--Classification of Cavities, by S. H. Guilford; Exclusion of Moisture—Ejection of the Saliva—Application of the Dam in Simple Cases, and in Special Cases presenting Difficult Complications—Napkins and other Methods for securing Dryness, by Louis Jack, The Selection of Filling Materials with reference to Character of Tooth Structure, Various Oral Conditions and Location, Depth of Cavity and Proximity of the Pulp—Cavity Lining, with its Purposes, by Louis Jack; Treatment of Fillings with respect to Contour, and the Relation of Contour to Preservation of the Integrity of Approximal Surfaces, by S. H. Guilford; The Operation of Filling Cavities with Metallic Foils and their Several Modifications, by E. T. Darby; Plastic Filling Materials—Their Properties, Uses and Manipulation, by H. H. Burchard; Combination Fillings, by D. M. Clapp; Inlays, by W. E. Christenson; The Conservative Treatment of the Dental Pulp, by Louis Jack; The Treatment and Filling of Root Canals, by H. H. Burchard; Dental-Alveolar Abscess, by H. H. Burchard; Pyorrhea Alveolaris, by C. N. Peirce; Discolored Teeth and Their Treatment, by E. C. Kirk; Extraction of Teeth, by M. H. Cryer; Extraction of Teeth under Nitrous Oxid Anesthesia, by J. D. Thomas; Local Anesthetics and Tooth Extraction, by Louis Ottofy; Management of the Deciduous Teeth, by C. L. Goddard; Orthodontia Exclusively as an Operative Procedure, by C. L. Goddard; The Development of Esthetic Facial Contours, by C. S. Case.

This work cannot fail to please the most fastidious, and must become a very popular treatise and is a worthy companion of the American Text Book of Prosthetic Dentistry.

